# Genome-Wide Divergence and Linkage Disequilibrium Analyses for *Capsicum baccatum* Revealed by Genome-Anchored Single Nucleotide Polymorphisms

**DOI:** 10.3389/fpls.2016.01646

**Published:** 2016-11-03

**Authors:** Padma Nimmakayala, Venkata L. Abburi, Thangasamy Saminathan, Aldo Almeida, Brittany Davenport, Joshua Davidson, C. V. Chandra Mohan Reddy, Gerald Hankins, Andreas Ebert, Doil Choi, John Stommel, Umesh K. Reddy

**Affiliations:** ^1^Department of Biology, Gus R. Douglass Institute, West Virginia State UniversityInstitute, WV, USA; ^2^Genetic Resources and Seed Unit, Asian Vegetable Research and Development Center-The World Vegetable CenterTainan, Taiwan; ^3^Department of Plant Science, Plant Genomics and Breeding Institute, College of Agriculture and Life Sciences, Seoul National UniversitySeoul, South Korea; ^4^Genetic Improvement of Fruits and Vegetables Laboratory (United States Department of Agriculture, Agricultural Research Service)Beltsville, MD, USA

**Keywords:** population structure, linkage disequilibrium, haplotyping, genotyping by sequencing, genome-wide association mapping, peduncle length

## Abstract

Principal component analysis (PCA) with 36,621 polymorphic genome-anchored single nucleotide polymorphisms (SNPs) identified collectively for *Capsicum annuum* and *Capsicum baccatum* was used to characterize population structure and species domestication of these two important incompatible cultivated pepper species. Estimated mean nucleotide diversity (π) and Tajima's D across various chromosomes revealed biased distribution toward negative values on all chromosomes (except for chromosome 4) in cultivated *C. baccatum*, indicating a population bottleneck during domestication of *C. baccatum*. In contrast, *C. annuum* chromosomes showed positive π and Tajima's D on all chromosomes except chromosome 8, which may be because of domestication at multiple sites contributing to wider genetic diversity. For *C. baccatum*, 13,129 SNPs were available, with minor allele frequency (MAF) ≥0.05; PCA of the SNPs revealed 283 *C. baccatum* accessions grouped into 3 distinct clusters, for strong population structure. The fixation index (*F*_*ST*_) between domesticated *C. annuum* and *C. baccatum* was 0.78, which indicates genome-wide divergence. We conducted extensive linkage disequilibrium (LD) analysis of *C. baccatum* var. *pendulum* cultivars on all adjacent SNP pairs within a chromosome to identify regions of high and low LD interspersed with a genome-wide average LD block size of 99.1 kb. We characterized 1742 haplotypes containing 4420 SNPs (range 9–2 SNPs per haplotype). Genome-wide association study (GWAS) of peduncle length, a trait that differentiates wild and domesticated *C. baccatum* types, revealed 36 significantly associated genome-wide SNPs. Population structure, identity by state (IBS) and LD patterns across the genome will be of potential use for future GWAS of economically important traits in *C. baccatum* peppers.

## Introduction

Chile peppers (*Capsicum* spp.) are represented by at least 32 species, of which *Capsicum annuum, Capsicum baccatum* L. var. *pendulum* (Willd.) Eshbaugh, *Capsicum chinense* Jacq., *Capsicum frutescens* L., and *Capsicum pubescens* Ruiz & Pavon represent domesticated taxa (Heiser and Smith, 1953; Eshbaugh, 1980; Pickersgill, 1991; Bosland and Votava, 1999; Chiou and Hastorf, 2014). The eastern slopes of highland Bolivia are considered the origin of the *Capsicum* genus, which spread through the pre-Holocene Americas via dispersal by birds or through river flows. *C. baccatum*, with yellow spotted white flowers, is thought to have domesticated in lowland Bolivia or coastal Peru, whereas entirely white-flowered *C. annuum* was domesticated in Mexico (Eshbaugh, 1980; Andrews, 1984; Pickersgill, 1997; Aguilar-Meléndez et al., 2009b; Chiou and Hastorf, 2014). Within the *C. baccatum* complex, *C. baccatum* var. *baccatum* and *C. baccatum* var. *pendulum* represent the wild and domesticated forms of the species, respectively. *C. baccatum* var. *pendulum* extends northwards to Ecuador and southern Colombia and eastwards to south-eastern Brazil (Pickersgill, 1971).

Pepper germplasm is a valuable resource for investigating the still-unresolved question of whether similar domestication related changes occurred independently to result in parallel or convergent evolution in the domestication syndrome (Pickersgill, 2007). Because *C. annuum* and *C. baccatum* are sexually incompatible, the question cannot be resolved by crossing these genetically isolated domesticated peppers. However, genomic tools offer a plethora of opportunities to compare domestication footprints and determine whether complementary or different loci are involved (Pickersgill, 2007). *C. baccatum* var. *pendulum* is known for great variability in fruit quality traits, yield, pathogen resistance, and bioactive compounds (Yoon et al., 2006; Rodríguez-Burruezo et al., 2009; Do Rêgo et al., 2009; Eggink et al., 2014). Conventional plant breeding programs require costly investments in time, labor and land to develop improved cultivars; the application of genomic tools combined with next-generation sequencing could accelerate the genetic improvement of peppers. The use of *C. baccatum* and *C. annuum* species in interspecific breeding programs has been limited because of post-fertilization barriers.

Several studies mainly explored genetic distances and phylogenetic analysis in *C. annuum* (Lefebvre et al., 1993; Prince et al., 1995; Paran et al., 1998; Livingstone et al., 1999; Rodriguez et al., 1999; Patricia Toquica et al., 2003; Kim and Kim, 2005; Lefebvre, 2005; Portis et al., 2007; Aguilar-Meléndez et al., 2009a; Mimura et al., 2012; Hill et al., 2013; Nicolaï et al., 2013; González-Pérez et al., 2014). We have only a few reports of the genetic diversity and population structure of *C. baccatum* var. *pendulum* (Albrecht et al., 2011, 2012; Ibiza et al., 2012).

Genotyping by sequencing (GBS) is a reduced representation method, which utilizes next-generation sequencing to develop genome-wide single nucleotide polymorphisms (SNPs). SNPs generated by GBS have been successfully deployed for genetic diversity analysis and Genome-wide association studies (GWAS) in several crops (Poland and Rife, 2012; Narum et al., 2013; Liu et al., 2014; Nimmakayala et al., 2014, 2016; Guajardo et al., 2015; Otto et al., 2016). Increased marker density across the chromosomes facilitates to estimate genome-wide non-random association of allelic states across the chromosomes, which is known as Linkage disequilibrium (LD; Mackay and Powell, 2007; Reddy et al., 2014; Baird, 2015; Wang et al., 2015; Zanke et al., 2015). GWAS models are to scan genome-wide LD blocks to identify causal locus for trait of the interest, while involving population structure and identity by state (IBS) matrices as the cofactors to reduce spurious associations due to confounding effects of population stratification and polygenic background (Rafalski, 2010; Stich and Melchinger, 2010; Newell et al., 2011). The availability of genome-wide (SNPs) affords new opportunities in the current study to better resolve *C. baccatum* population structure, LD and diversity and dissect the population demographic history across the genome by comparison with another domesticated species, *C. annuum*. In addition, we utilized population structure analyses for a genome-wide association study (GWAS) of peduncle length, an important domestication trait.

## Materials and methods

### Germplasm

A representative sample of 377 pepper accessions (283 *C. baccatum* and 94 diverse *C. annuum* accessions) collected from 32 countries across the world were obtained from the USDA-ARS, Germplasm Resource Information Network, Plant Genetic Resources Conservation Unit, Griffin, GA and World Vegetable Center (AVRDC, Shanhua, Taiwan) (Table S1). The *C. annuum* collection was comprised of 90 domesticated cultivars and 4 wild accessions. The *C. baccatum* collection had 218 lines of *C. baccatum* var. *pendulum* and 17 wild accessions (*C. baccatum var. baccatum*). Peduncle length (cm) was measured for 5 plants each of 217 accessions belonging to *C. baccatum* var. *pendulum* grown in a greenhouse in three replications.

### Genotyping by sequencing (GBS)

Genomic DNA was isolated from the seedlings using the DNeasy plant mini kit (QIAGEN, Germany), and GBS was as described (Elshire et al., 2011). DNA was treated with the restriction enzyme ApeKI, a type II restriction endonuclease, barcoded by accession, and sequenced on an Illumina HiSeq 2500 as described (Elshire et al., 2011). SNPs were identified using the TASSEL-GBS Discovery/Production pipeline (https://bitbucket.org/tasseladmin/tassel-5-source/wiki/Tassel5GBSv2Pipeline). Chromosomal assignment and position on the physical map of various SNPs were deduced from the *C. annuum* whole genome sequence (WGS) draft at http://peppergenome.snu.ac.kr. SNPs were designated by chromosome number and position (e.g., S10_172735351, which indicates an SNP located at position 172735351 on chromosome 10).

### Genome-wide divergence and population structure analysis

Genetic diversity values were calculated by a neighbor-joining algorithm using TASSEL 5. In a second approach, we utilized IBS and principle component analysis (PCA) with the SNP & Variation Suite (SVS v8.1.5) (Golden Helix, Inc., Bozeman, MT, USA; www.goldenhelix.com). Observed nucleotide diversity (π) and Tajima's D were estimated by using TASSEL v5.0 with a sliding-window approach as described (Korneliussen et al., 2013). The fixation index (*F*_*ST*_) was estimated on the basis of the Wright F statistic (Weir and Cockerham, 1984) with use of SVS v8.1.5.

### Characterization of linkage disequilibrium (LD)

For GBS data, we considered only SNPs successfully mapped to the *C. annuum* WGS draft, because knowing the chromosome location of SNPs helps prevent spurious LD and thereby unreliable association mapping. Mapped SNPs were further filtered by call rate >90%. Before studying LD decay, haplotype blocks were calculated for all markers by using the default settings in SVS v8.1.5. Adjacent and pairwise measurements of LD for GBS data were calculated separately for SNPs in each chromosome. For computing LD, we used the expectation-maximization (EM) algorithm (Dempster et al., 1977) as an iterative technique for obtaining maximum likelihood estimates of sample haplotype frequencies.

### GWAS mapping

The PC matrix was constructed with the program “EIGENSTRAT” (http://genetics.med.harvard.edu/reich/Reich_Lab/) and the PCA correction technique; the method of stratification was as described (Price et al., 2006). IBS was calculated as described (Purcell et al., 2007). GWAS involved a single-locus mixed linear model (SLMM), a method that uses a forward and backward stepwise approach to select markers as fixed-effects covariates in the model (Segura et al., 2012), and implemented in SVS v8.1.5. We used a PC matrix to correct for population stratification and an IBS matrix to correct for a polygenic background. Manhattan plots for associated SNPs were visualized by using GenomeBrowse v1.0 (Golden Helix, Inc.). The SNP *P*-values from GWAS underwent false discovery rate (FDR) analysis (Storey, 2002).

## Results

### SNP development

A total of 77,407 SNPs were isolated from the nucleotide sequence obtained for the 283 *C. baccatum* and 94 *C. annuum* accessions studied; a total of 8661, 8086, 9843, 6197, 5688, 7410, 5588, 5086, 4472, 5336, 5079, and 5961 SNPs were mapped to the WGS draft and located on chromosomes 1, 2, 3, 4, 5, 6, 7, 8, 9, 10, 11, and 12, respectively. We noted the presence of one SNP at every 35.6 kb across the genome, with average gap size of 31.7 kb and one SNP at every 104.4 kb in the coding regions. A total of 36,621 SNPs had minor allele frequency [MAF] ≥0.05, identified collectively for *C. annuum* and *C. baccatum*, and were used for various analyses in the current study. For *C. baccatum*, 13,129 SNPs had MAF ≥0.05; their chromosome distribution is listed in Table 1. In addition, we identified 26,697 SNPs located in various exons. SNP counts in exons of various genes were 2985, 3308, 3630, 2032, 1837, 2474, 1897, 1758, 1406, 1799, 1550, and 2021 on chromosomes 1, 2, 3, 4, 5, 6, 7, 8, 9, 10, 11, and 12, respectively.

**Table 1:** **Chromosome-wise distribution 13,129 SNPs with MAF of ≥0.05 for *C. baccatum* collections**.

**Chromosome number**	**No. of SNPS**
1	1443
2	1220
3	1447
4	1259
5	1198
6	1302
7	844
8	752
9	752
10	970
11	922
12	1020
Total	13,129

### Population stratification

**Figure 1:**
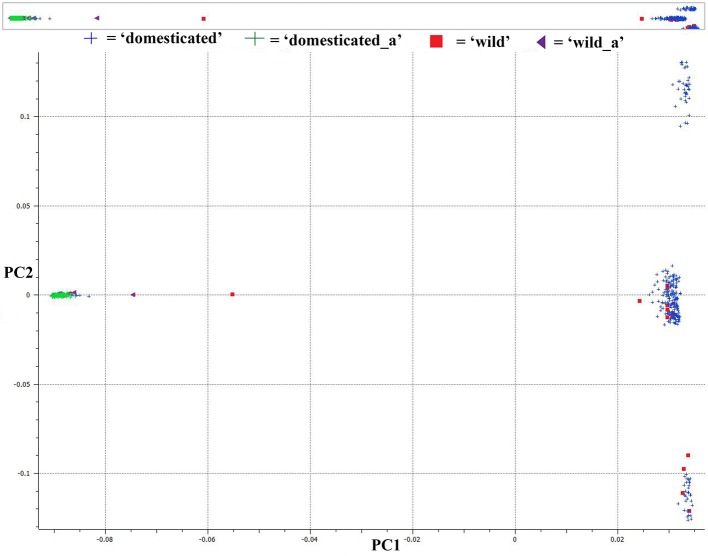
**First and second principal component analysis (PCA) components for 36,621 single nucleotide polymorphisms (SNPs) in a set of 377 diverse pepper accessions (283 *Capsicum baccatum* and 94 *C. annuum* accessions)**. See Table S2 for a list of accessions and eigen values for respective positions of individual accessions in the figure.

**Figure 2:**
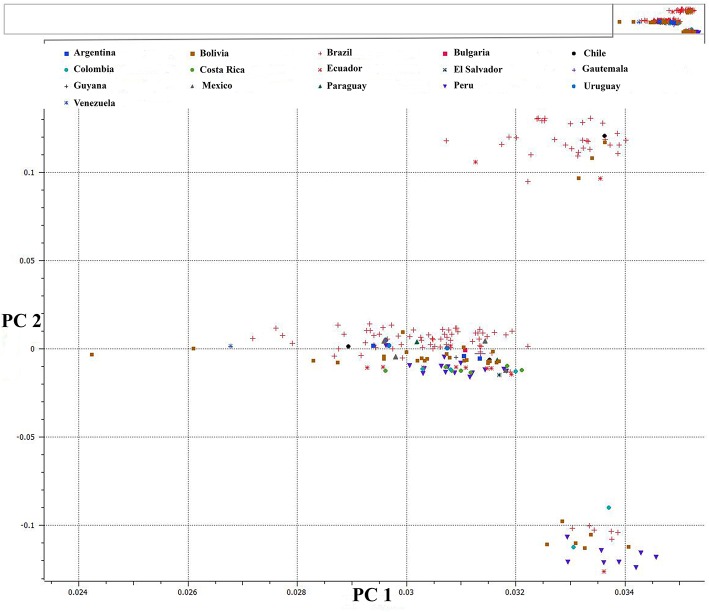
**First and second PCA components for 13,129 SNPs within 283 *C. baccatum* accessions**. See Table S3 for a list of accessions and eigen values for respective positions of individual accessions in the figure.

We used PCA of the 36,621 SNPs identified from *C. baccatum* and *C. annuum* with MAF ≥0.05 to characterize domesticated and wild *C. annuum* and *C. baccatum* peppers. PCA with first and second eigen vectors that explained 80% of the total variation produced two clusters of *C. baccatum* and *C. annuum* accessions (Figure 1). Tepin and Tepin Guatemala, two wild peppers belonging to *C. annuum* var. *glabriusculum* that are native to southern North America and northern South America, were close to CB-77, a wild *C. baccatum* pepper. Similarly, three other wild *C. baccatum* peppers, CB-93, CB-92, and CB-40, were intermediate between the major *C. annuum* and *C. baccatum* clusters. A third cluster comprised the remaining wild, semi-domesticated and crown shaped fruit type *C. baccatum* accessions that grouped with the domesticated large-fruited *C. baccatum* peppers. A separate PCA with 13,129 SNPs that were polymorphic for *C. baccatum* accessions resolved the population structure comprised by this group of *C. baccatum* accessions. This PCA identified 283 *C. baccatum* accessions in 3 distinct clusters (Figure 2). The middle cluster (cluster II) was parallel to the *C. annuum* cluster, and the wild species Tepin, Tepin Guatemala, CB-77, CB-93, CB-92, and CB-40 were found in the middle, which indicates intercrossing between wild *C. annuum* and *C. baccatum* peppers while or before domestication. PCA placement of various accessions are noted in Tables S2, S3.

### Fixation index (*F_*ST*_*) distribution to locate positive selection footprints

**Figure 3:**
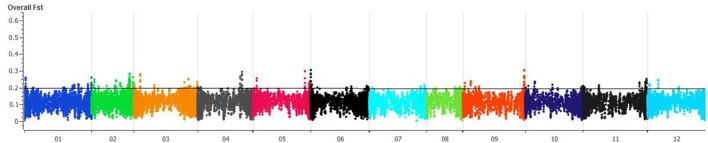
**Manhattan plot of chromosome-wise overall fixation index (*F*_*ST*_) values for 283 *C. baccatum* accessions**. Individual *F*_*ST*_-values are in Table S4.

*F*_*ST*_ was estimated with 95% confidence intervals between wild and domesticated *C. annuum* and *C. baccatum*. The *F*_*ST*_ between wild (*C. annuum* + *C. baccatum*) and domesticated (*C. annuum* + *C. baccatum*) accessions was 0.09 and 0.05, respectively. The *F*_*ST*_between domesticated *C. annuum* and *C. baccatum* was 0.78, which indicates genome-wide divergence. The *F*_*ST*_ between wild *C. baccatum* and wild *C. annuum* was 0.66. Crown-shaped fruited *C. baccatum* types are unique for this species group, and pairwise *F*_*ST*_ values with wild, semi-domesticated and domesticated were 0.10, 0.06, and 0.03, respectively, which indicates their closeness to domesticated types. *F*_*ST*_-values for semi-domesticated with wild and domesticated *C. baccatum* types were 0.03 and 0.01, respectively. We present an overall *F*_*ST*_ distribution in a Manhattan plot for all chromosomes showing important chromosomal regions with the highest *F*_*ST*_ as peaks (Figure 3, Table S4). Based on *F*_*ST*_ values, peaks on chromosomes 1, 2, 3, 4, 5, 6, and 9 in the Manhattan plot might be the regions of positive selection and important for improvement.

**Figure 4:**
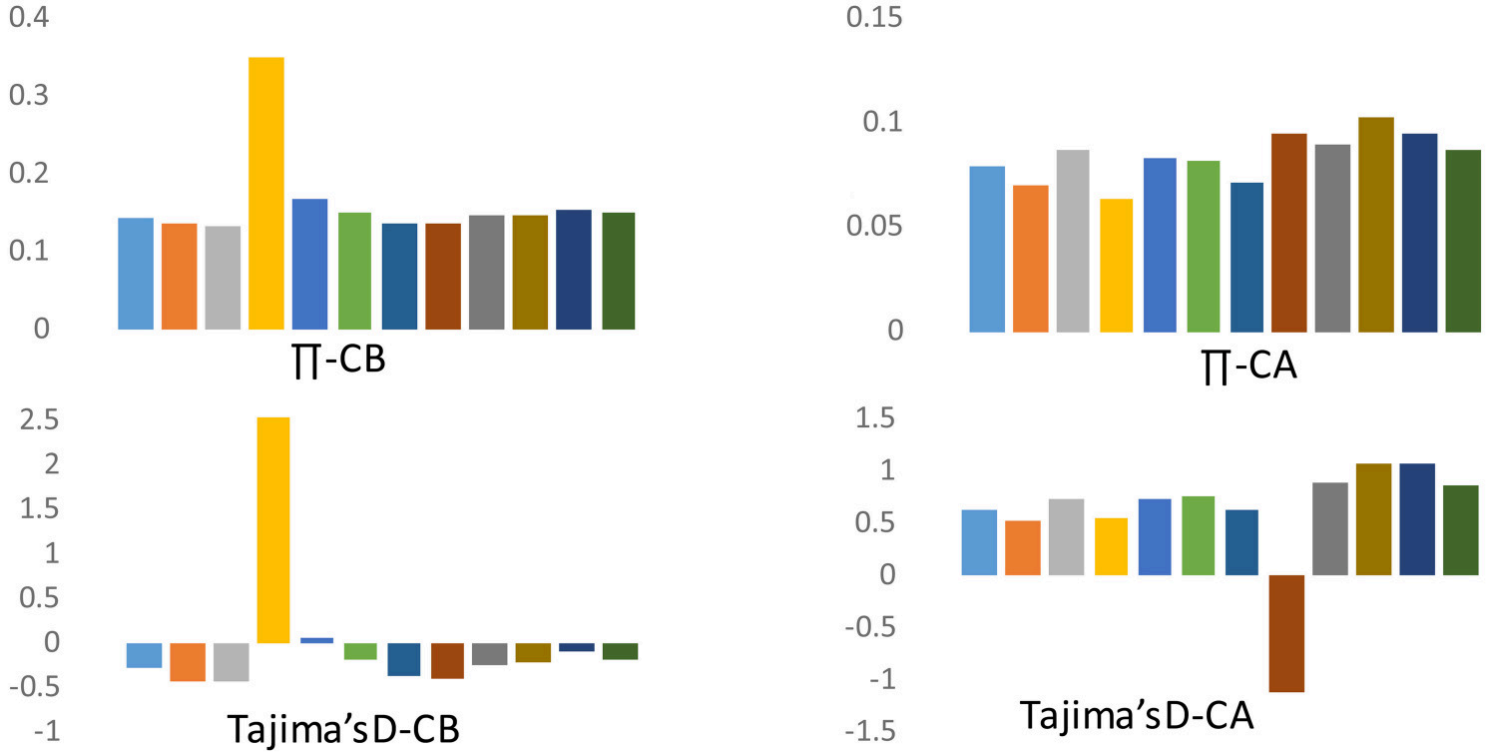
**Frequency spectrum for chromosomal means for nucleotide diversity (π) and Tajima's D for *C. annuum* (CA) and *C. baccatum* (CB) domesticated accessions**.

Because of the strong population structure, we assessed patterns of variation separately for each group of domesticated accessions from the respective species when making inferences about the evolutionary dynamics of domestication. Crop domestication is often associated with “population bottlenecks” because of the limited number of founding individuals experiencing domestication events. These bottlenecks may be evident in pepper when comparing diversity between cultivated forms of *C. annuum* and *C. baccatum*. We estimated nucleotide diversity (π) and Tajima's D across various chromosomes to understand genome-wide bottleneck effects. The frequency of segregating SNPs as reflected by various chromosomal measures of mean π and Tajima's D is presented in Figure 4. For cultivated *C. baccatum*, chromosome 4 was positive for π and Tajima's D which indicates accumulation of rapid mutations on this chromosome. The remaining chromosomes were negative or nearly negative for Tajima's D, which indicates bottlenecks in domestication. In contrast, *C. annuum* chromosomes were positive for Tajima's D on all chromosomes except chromosome 8, which indicates differential evolution after the domestication or the influence of diverse breeding.

### LD analysis for *C. baccatum*

**Figure 5:**
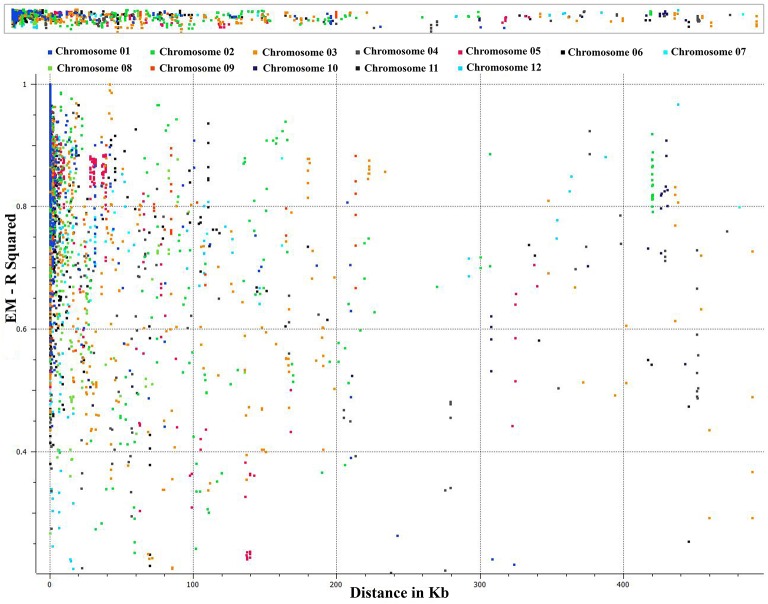
**Genome-wide distribution of marker associations (*r*^2^) based on expectation-maximization (EM) analysis for adjacent SNPs across chromosomes showing most SNP associations (LD) decay within 50 kb**.

We conducted an extensive LD analysis on the entire dataset of *C. baccatum* accessions on all adjacent marker pairs within a chromosome or within a haplotype block. Haplotype distribution is important to understand patterns of genetic variation of *C. baccatum* gene pools and has a wide range of applications. The 2 major processes that shape haplotype structure are the domestication process and breeding history. We used “minimize historical recombination,” a block-defining algorithm developed by Gabriel et al. (2002). The upper confidence boundary was set to 0.98 and the lower boundary to 0.70. SNPs with MAF <0.05 were omitted. Maximum block length was set to 160 kb. The expectation maximization (EM) algorithm was used for haplotype estimation, with convergence tolerance 0.0001, and frequency threshold 0.01. Maximum EM iterations were set to 50. We identified 1742 haplotypes containing 4420 SNPs, with a range of 9–2 SNPs per haplotype (Table S5). The results provided values for both the EM algorithm (Dempster et al., 1977) and composite haplotype method (CHM; Weir and Cockerham, 1996). Squared-allele frequency correlations (*r*^2^) and LD estimate (D′) for the EM and CHM methods are in Table S6. We created LD plots by using marker-pair associations of adjacent SNPs within a chromosome, within a haplotype block, and within genes (Figure 5). The length of individual LD blocks varied among chromosomes, with regions of high and low LD interspersed (Table 2). The genome-wide average LD block was 99.1 kb. The largest LD block, of 13,021 kb, was on chromosome 11. Pairwise LD was estimated by *r*^2^ and we compared the pattern of decay at different levels. With pair-wise analysis considering adjacent SNPs across chromosomes, most SNP associations were within 50 kb (Figure 5). The second analysis based on adjacent SNPs within haplotypes revealed most associations within 20 kb (Figure S1). The third analysis of SNPs located in genes revealed most associations within 5 kb (Figure S2).

**Table 2:** **Chromosome-wise distribution of LD blocks for *C. baccatum* var. *pendulum***.

**Chromosome** **number**	**LD analysis with adjacent SNPs**		
	**No. of SNP associations**	**Mean LD block size (Kb)**	**Maximum LD block size (Kb)**
1	721	71.861	3948.923
2	636	91.05	10856.72
3	756	67.397	3122.154
4	603	88.686	7216.827
5	670	110.614	6046.404
6	670	88.726	4043.506
7	390	90.642	4527.862
8	400	96.781	6962.39
9	401	169.852	4569.492
10	471	138.615	8694.085
11	494	117.027	13021.65
12	533	104.171	7352.753
Overall	6745	99.11	13021.65

### GWAS for peduncle length

**Figure 6:**
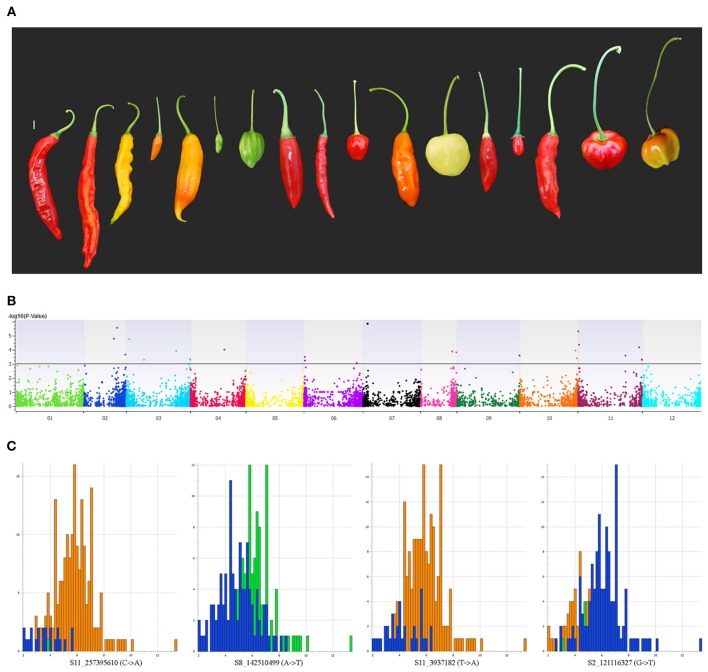
**Manhattan plot of the genome-wide association study for peduncle length in *C. baccatum* var. *pendulum*. (A)** Range of observed peduncle length. **(B)** Chromosome coordinates are on the X-axis, with the negative log-10 of the association *P*-value for each SNP on the Y-axis. High negative log-10 indicates strong association with the trait. Histograms show effects of significantly associated SNPs for peduncle length. **(C)** Four SNPs located in the intergenic space between the oxidoreductase family protein/arogenate dehydrogenase on chromosome 7 that explained 10.6% of the total variation for peduncle length.

Peduncle length is the prime differentiating trait between wild and domesticated forms of *C. baccatum*. Mean peduncle lengths for respective accessions are listed in Table S7. The cultivated form of *C. baccatum*, var. *pendulum*, is named based on the epithet related to pendant fruits. In our GWAS, 36 SNPs located on chromosomes 1, 2, 3, 4, 6, 7, 8, 9, 10, and 11 were identified as significantly associated with peduncle length and cumulatively explained 21% of the total variation (Figure 6). Four SNPs located in the intergenic space between the oxidoreductase family protein/arogenate dehydrogenase on chromosome 7 explained 10.6% of the total variation. Chromosome number, map position, *P*-value, regression beta, FDR correction, variance explained, call rate, and minor/major allele frequencies for all significantly associated SNPs are in Table S8.

### Candidate gene selection

The predicted gene set from the annotated *C. annuum* cv. CM334 reference genome (Kim et al., 2014) was used to characterize the genes containing SNPs or nearby SNPs. Eleven candidate genes containing SNPs in exons or promoters were significantly associated with peduncle length, and 12 more SNPs in introns or intergenic regions of candidate genes were proposed. GWAS details and strengths of association of SNPs are in Table S8. Details of annotation for various associated SNPs, their location in various genes and type of mutation (synonymous or non-synonymous) are in Table 3.

**Table 3:** **Annotation of significantly associated SNPs for peduncle length in *C. baccatum* var. *pendulum***.

**Marker**	***P*-Value**	**−log10(*P*-Value)**	**FDR**	**Locus ID**	**Location**	**Ma → Mi**	**Sy/NSy**	**Annotation/Function**
S7_19145046	1.13E−06	5.947	0.015	CA07g03460/CA07g03470	Intergenic	G → C	–	Oxidoreductase family protein/Arogenate dehydrogenase
S7_19145048	1.13E−06	5.947	0.007	”	”	C → T	”	Oxidoreductase family protein/Arogenate dehydrogenase
S7_19145066	1.13E−06	5.947	0.005	”	”	T → A	”	Oxidoreductase family protein/Arogenate dehydrogenase
S7_19145073	1.13E−06	5.947	0.004	”	”	C → T	”	Oxidoreductase family protein/Arogenate dehydrogenase
S2_134518344	3.89E−06	5.410	0.010	CA02g11490	Exon	G → C	R → P^*^	Phospho-n-acetylmuramoyl-pentapeptide-transferase
S11_725918	7.48E−06	5.126	0.016	CA11g00270/CA11g00280	Intergenic	C → G	–	GABA-specific permease/Unknown protein
S2_121116327	2.01E−05	4.697	0.038	CA02g09090	Exon	G → T	T → K^*^	LON peptidase N-terminal domain and RING finger protein
S3_12740983	2.22E−05	4.653	0.036	CA03g04980	Intron	C → T	–	Eukaryotic translation initiation factor 2 subunit alpha
S11_190326151	3.12E−05	4.505	0.046	CA11g12020	Exon	G → A	S → S	Tho2 protein
S11_3937182	5.13E−05	4.289	0.061	CA11g01740	Exon	T → A	Q → L^*^	Hydroxyproline-rich glycoprotein
S3_200716267	6.71E−05	4.173	0.073	CA03g17680	Intron	A → C	–	Pre-mRNA cleavage factor IM
S11_246730373	0.0001	3.916	0.122	CA11g15960	Intron	G → A	–	ATP-dependent RNA helicase
S4_137196865	0.0001	3.864	0.128	CA04g10860/CA04g10870	Intergenic	C → T	–	Amino acid transporter/UDP-glucose 6-dehydrogenase
S4_137196912	0.0001	3.864	0.120	”	”	C → A	”	Amino acid transporter/UDP-glucose 6-dehydrogenase
S10_223493543	0.0002	3.807	0.128	CA10g17500/CA10g17510	Intergenic	C → G		Cytochrome b559 subunit alpha/Aluminum-activated malate transporter
S8_126682716	0.0002	3.730	0.144	CA08g09170	Exon	C → T	G → R^*^	Ribosomal protein S11
S8_126682746	0.0002	3.730	0.136	”	”	C → A	A → S^*^	Ribosomal protein S11
S9_252073885	0.0004	3.449	0.195	CA09g18340	Promoter	G → A	–	Reticulon-like protein B21
S9_252073890	0.0004	3.449	0.187	”	”	G → A	”	Reticulon-like protein B21
S11_190326131	0.0004	3.449	0.180	CA11g12020	Exon	G → A	S → L^*^	Tho2 protein
S10_229515157	0.0004	3.412	0.188	CA10g19840	Exon	G → A	S → F^*^	Uncharacterized protein
S6_2635088	0.0004	3.381	0.195	CA06g01230/CA06g01240	Intergenic	T → C	–	Late blight resistance protein Rpi-blb2/Detected protein of confused Function
S8_142510499	0.0005	3.315	0.219	CA08g18030	Exon	A → T	M → L^*^	Serine/Threonine-protein kinase SMG1
S1_96976222	0.0005	3.285	0.227	CA01g16010	Exon	T → C	T → T	Phytochrome
S2_139076418	0.0005	3.263	0.231	CA02g13050	Intron	T → G	–	Ureidoglycolate hydrolase
S3_70295226	0.0006	3.220	0.247	CA03g11420/CA03g11430	Intergenic	C → T	–	Detected protein of confused Function/NADH dehydrogenase subunit
S3_257225287	0.0006	3.188	0.251	CA03g36710/CA03g36720	Intergenic	C → T	–	LRR receptor protein kinase/LRR receptor protein kinase
S11_257395608	0.0007	3.183	0.246	CA11g19730	Exon	C → T	H → H	ABC transporter
S11_257395610	0.0007	3.183	0.239	”	”	C → A	A → D^*^	ABC transporter
S2_130946711	0.0007	3.180	0.234	CA02g10590/CA02g10600	Intergenic	C → T	–	Nucleic acid binding protein/cleavage and polyadenylation specificity factor CPSF30
S3_252341359	0.0008	3.078	0.289	CA03g33810	Intron	T → G	–	DNase I-like superfamily protein
S1_131644198	0.0009	3.046	0.303	CA01g17480	Promoter	A → G	–	Diacylglycerol kinase variant B

* Nonsynonymous mutation on amino acid due to minor/major allele SNP variation.

## Discussion

The cultivated pepper species, *C. baccatum*, known as aji or Peruvian hot pepper, is a valuable source of novel genes that has not yet been analyzed for genome-wide diversity and population structure (Albrecht et al., 2012). Our genome-wide diversity analysis showed that many domesticated *C. baccatum* var. *pendulum* from western Bolivia/Peru and eastern Brazil/Paraguay cluster with most wild-type *C. baccatum* var. *baccatum*, suggesting that they may be the ancestral cluster. The flow of the river Rio Mizque from the south to join the Amazon is through lowland tropical Bolivia and the Amazon Basin and thus includes both the range of the *C. baccatum* group and a portion of the range of the *C. annuum* group (Eshbaugh, 1980). McLeod et al. (1982) suggested that the white-flowered ancestor migrated to dry areas of southern Bolivia, to produce the *C. baccatum* group, and the wild form in the wetter Amazon basin developed into the wild progenitor for *C. annuum*.

Our comparative divergence analysis across the chromosomes for *C. annuum* and *C. baccatum* revealed that chromosome 4 of *C. baccatum* had a unique divergence history, and for *C. annuum*, chromosome 8 showed a differential evolution when comparing mean π and Tajima's D for various chromosomes. In addition, biased distribution of Tajima's D toward negative values on all chromosomes (except chromosome 4) in cultivated *C. baccatum* indicates a population bottleneck during domestication or through the breeding histories, or the speciation of *C. baccatum* might have occurred with relatively narrow genetic diversity. In contrast, *C. annuum* chromosomes showed positive Tajima's D on all chromosomes except chromosome 8, which indicates that speciation or domestication of *C. annuum* might have occurred at multiple sites, contributing to wider genetic diversity as discussed by Kraft et al. (2014). Subsequent spread of *C. annuum* cultivars across the world and exposure to diverse breeding programs or selection in conjunction with diverse ecological adaptation might explain such rapid population size expansion and recovery from the bottleneck effects. The genome size of *C. annuum* types was estimated to be 3691 Mbp and *C. baccatum* was 4048 Mbp, which indicates wide divergence between these 2 cultivated pepper genomes (Belletti et al., 1998). Tang et al. (2006) concluded that unusually divergent genomic regions between closely related rice species are informative about species incompatibility or reproductive barriers resulting in partial fertility. Similar to the current findings, several reports implicated newly recruited polymorphisms as causing highly divergent genomic regions that may control traits associated with reproductive incompatibility or ecological adaptation (Wu, 2001; Wu and Ting, 2004).

Current advances in genome sequencing for identifying genome-wide SNPs and mapping them to WGS drafts allowed for scanning of LD decay across the genome. LD, the non-random association of alleles at different loci and germplasm panels that represent genome-wide cultivar diversity (power of association panel), plays an integral role in GWAS and determines the density of SNPs required for GWAS (Flint-Garcia et al., 2003; Nicolas et al., 2016). Low to moderate LD (decay within 100 kb) such as that observed for the *C. baccatum* panel in our study must utilize high SNP density (Kovi et al., 2015). In this study, we noted the highest LD for chromosome 11. One explanation for such variable LD is the “Bulmer effect,” whereby high LD regions are generally associated with selective sweeps harboring important genes underlying domestication (Bulmer, 1971; Kovi et al., 2015). The stochastic process that generates LD during selective sweeps is because of a spontaneous mutation leading to an advantageous effect or LD decays with recombination with a diverse haplotype and further segregation (Baird, 2015).

### GWAS for peduncle length

Wild *C. baccatum* has a relatively restricted distribution confined to southern Peru, Bolivia, and southern Brazil (Eshbaugh, 1970). *C. baccatum* var. *pendulum* is a widely distributed cultivated plant found throughout western South America and now spreading worldwide (Eshbaugh, 1970). Wild *C. baccatum* has red, erect, and non-persistent fruits, and *C. baccatum* var. *pendulum* has red, orange, yellow, green, or brown fruits that are pendant and persistent. Because the peduncle is the most differentiating trait between domesticated and wild *C. baccatum* species, we performed GWAS for peduncle length. We associated 36 SNPs with the trait peduncle. Four of these SNPs clustered with candidate genes on chromosome 7. Annotation for some of these associated SNP-containing sequences revealed their location in various genes, so these genes might play a role in peduncle length, peduncle architecture and *C. baccatum* domestication.

Length of peduncle is determined by the cell number or cell size, although it is indirectly regulated by hormones and multiple pathways. Kinases play important roles in plant growth and development. Peduncle associated SNPs in the current study were located in leucine-rich repeat receptor like kinases (LRR-RLKs), serine/threonine protein kinase, ABC transporter gene and RING finger protein, which may play important roles in growth and development as well as cell wall integrity and elongation as has been shown in other plants (Lally et al., 2001; Arunyawat et al., 2007; Guo et al., 2009; Gish and Clark, 2011; Ghosh et al., 2013). Plant cell walls contain a glycoprotein component rich in the otherwise rare amino acid hydroxyproline and accumulation of this amino acid was positively correlated with cell elongation in pea epicotyls (Flint-Garcia et al., 2003). In the current study, we also associated a marker S11_725918 on GABA (γ-aminobutyric acid), a ubiquitous non-protein amino acid. An Arabidopsis GABA gene mutant *pop2* exhibited defects in hypocotyl cell elongation and pollen-tube elongation via influence on cell-wall–related genes (Bulmer, 1971).

Our study describes the utility of SNPs generated by GBS for genome-wide divergence and LD patterns between *C. annuum* and *C. baccatum*. Mapping all the SNPs to the *C. annuum* reference genome helped to identify homologous SNPs between the two incompatible cultivated pepper genomes, which was further useful to reduce ascertainment bias, so this SNP set was useful in estimating genome-wide population differentiation and allele sharing between the two genomes. Furthermore, the SNPs anchored to the *C. annuum* genome may not be in the same order in the *C. baccatum* genome because some genomic regions may not be co-linear to the *C. annuum* genome because of genome rearrangements. In a comparison of *C. baccatum* and *C. annuum* linkage maps, Lee et al. (2016) identified two major reciprocal translocations between chromosomes 3 and 5 and between chromosomes 3 and 9, as well as translocations between chromosomes 1 and 8.

Such uncertain positions of SNPs can be corrected only when the whole genomesequence is available for *C. baccatum* genome. This SNP panel and the results pertaining to population structure, IBS and LD decay analyses will facilitate routine use of GWAS for identification of genes associated with various economically important traits in Peruvian peppers. Our identification of SNPs associated with fruit peduncle length demonstrates opportunities for utilization of GWAS in crop improvement.

## Author contributions

UR, PN, JS, GH, and AE designed the study and drafted the manuscript. PN, VA, JD, and BD conducted peduncle phenotyping. PN, VA, AA, JD, and BD extracted DNA and assisted to generate genome-wide SNPs. DC provided whole genome sequence draft and mapped SNPs to the genome. UR, PN, CR, TS, AA, and VA performed population structure and GWAS analysis.

### Conflict of interest statement

The authors declare that the research was conducted in the absence of any commercial or financial relationships that could be construed as a potential conflict of interest.

## Supplementary material

Table S1 **List of *Capsicum annuum* and *C. baccatum* pepper accessions used in the current study**.

Table S2 **Eigen values for the first two components from principle component analysis (PCA) estimated for various accessions belonging to *C. annuum* and *C. baccatum***.

Table S3 **Eigen values for the first two PCA components estimated for various accessions belonging to *C. baccatum***.

Table S4 **Fixation index (*F*_*ST*_) values for individual SNPs across chromosomes of *C. baccatum* genome**.

Table S5 **Haplotype blocks across the cultivated *C. baccatum* genome**.

Table S6 **Linkage disequilibrium (LD) analysis of adjacent SNP pairs across the *C. baccatum* genome**.

Table S7 **Phenotypic data for mean peduncle length (cm) for 217 *C. baccatum* var. *pendulum* accessions**.

Table S8 **Details of significantly associated SNPs as revealed by genome-wide association study**.

Figure S1 **LD analysis (*r*^2^) based on adjacent SNPs within haplotypes showing most associations within 20 kb**.

Figure S2 **LD analysis (*r*^2^) of SNPs located in genes showing most associations within 5 kb**.
