# Supplementary figures and images for: Genome-Wide Divergence and Linkage Disequilibrium Analyses for Capsicum baccatum Revealed by Genome-Anchored Single Nucleotide Polymorphisms
Source: Front Plant Sci. 2016 Nov 3;7:1646. doi: 10.3389/fpls.2016.01646 (PMC5093146; doi:10.3389/fpls.2016.01646)

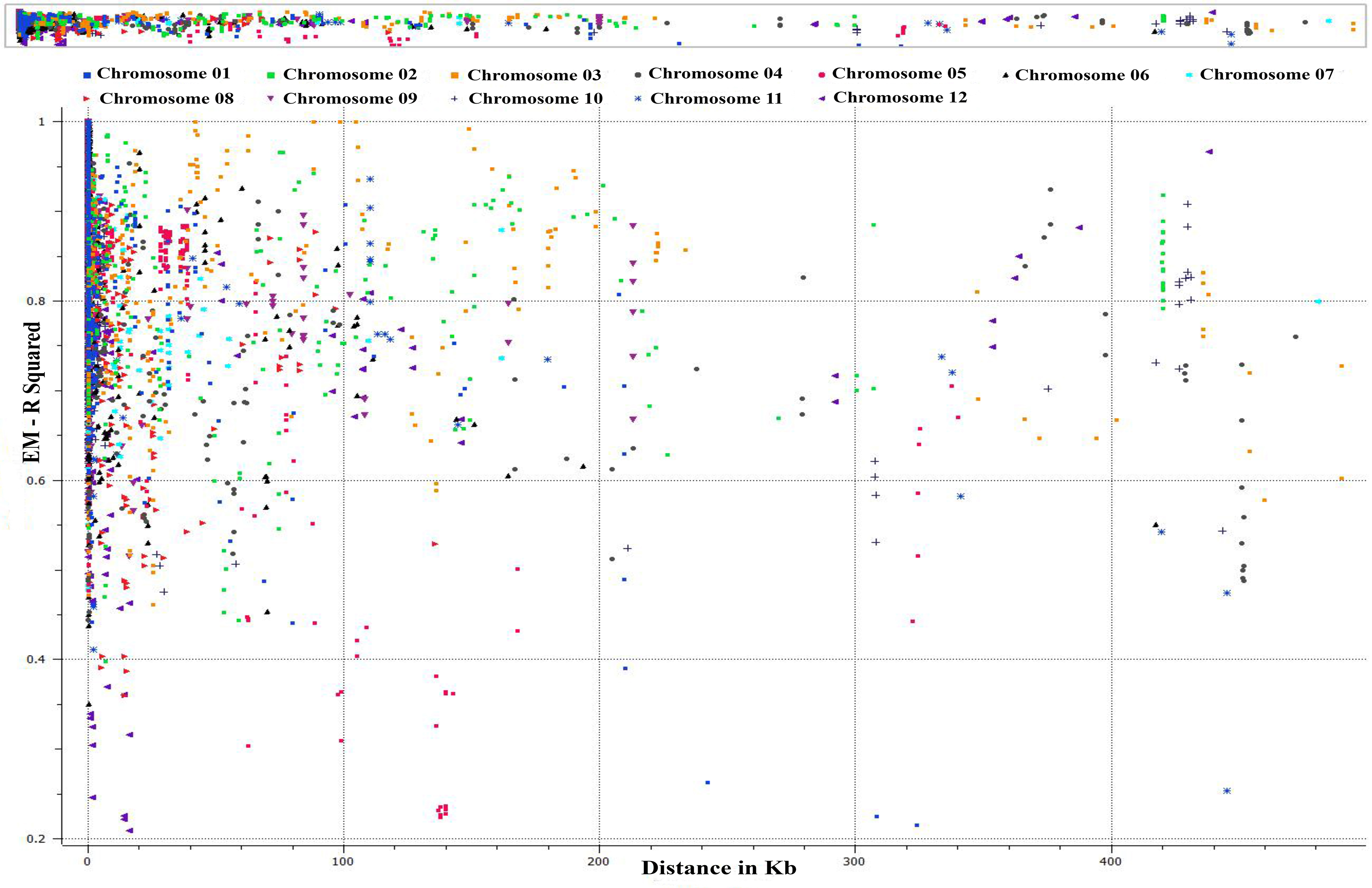

Supplement: Figure S1 — LD analysis (r2) based on adjacent SNPs within haplotypes showing most associations within 20 kb. [file Image1.JPEG]

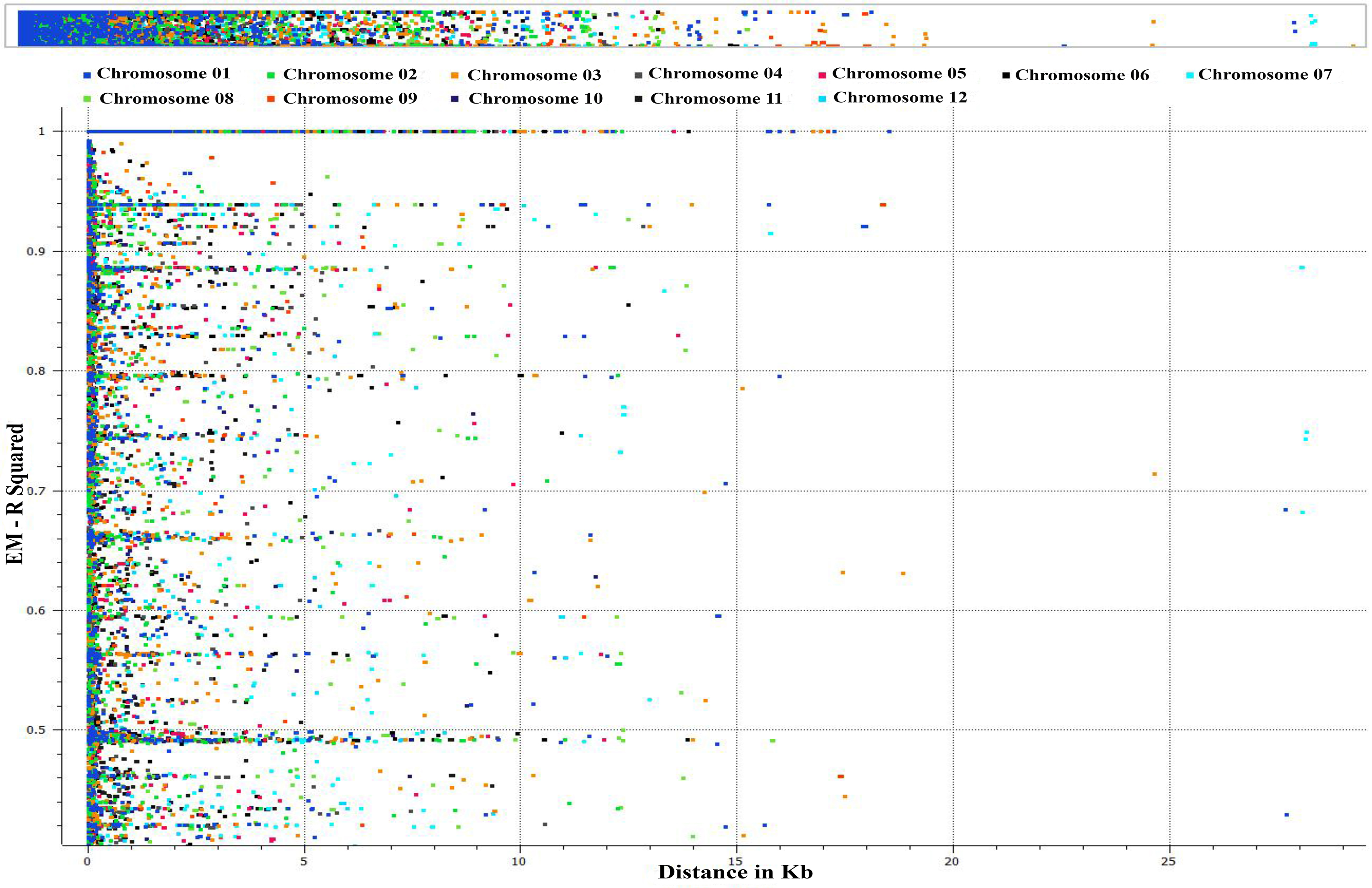

Supplement: Figure S2 — LD analysis (r2) of SNPs located in genes showing most associations within 5 kb. [file Image2.JPEG]
